# Reproductive factors, exogenous hormone use and incidence of melanoma among women in the United States

**DOI:** 10.1038/s41416-019-0411-z

**Published:** 2019-02-28

**Authors:** Grayson M. Donley, Wayne T. Liu, Ruth M. Pfeiffer, Emily C. McDonald, Kamau O. Peters, Margaret A. Tucker, Elizabeth K. Cahoon

**Affiliations:** Division of Cancer Epidemiology and Genetics, National Cancer Institute, National Institutes of Health, U.S. Department of Health and Human Services, Rockville, MD USA

**Keywords:** Melanoma, Cancer epidemiology

## Abstract

**Background:**

Although the photosensitising effects of oestrogens may increase the impact of ultraviolet radiation (UVR) on melanoma risk, few prospective studies have comprehensively assessed the association between oestrogen-related factors and melanoma.

**Methods:**

We examined the associations between reproductive factors, exogenous oestrogen use and first primary invasive melanoma among 167 503 non-Hispanic white, postmenopausal women in the NIH-AARP Diet and Health Study. Satellite-based ambient UVR estimates were linked to geocoded residential locations of participants at study baseline.

**Results:**

Increased risk of melanoma was associated with early age at menarche (≤10 vs ≥15 years: HR = 1.25, 95% CI: 0.92, 1.71; *P* for trend = 0.04) and late age at menopause (≥50 vs <45 years: HR = 1.34, 95% CI: 1.13, 1.59; *P* for trend = 0.001). The relationship between ambient UVR and melanoma risk was highest among women with age at menarche ≤10 years (HR per UVR quartile increase = 1.29; 95% CI: 1.05, 1.58; *P*-interaction = 0.02). Melanoma risk was not associated with parity, age at first birth, use of oral contraceptives or use of menopausal hormone therapy.

**Conclusions:**

Our findings suggest that increased melanoma risk is associated with early age at menarche and late age at menopause. Effect modification findings support the hypothesis that endogenous oestrogen exposure in childhood increases photocarcinogenicity. Future studies should include information on personal UVR exposure and sun sensitivity.

## Background

Cutaneous melanoma is the fifth most common cancer in the United States with an estimated 87,110 new cases and 9730 deaths expected in 2017.^1,2^ Well-established risk factors for melanoma include ultraviolet radiation (UVR) exposure,^3–5^ pigmentary traits,^6,7^ melanocytic nevi,^8,9^ family history of melanoma (including high- and low-risk susceptibility genes^6,10^), inherited genetic conditions^11^ and older age.^7,10^ The age-specific incidence of melanoma is slightly greater among women than among men in the US until the age of 50 years, at which point the rates of melanoma in men begin to increase sharply, while incidence rates in women tend to level off.^12^ The observation of these sex differences in the US and Europe,^13–15^ historical case reports of melanoma diagnosed during pregnancy,^16^ case reports of photosensitivity following oestrogen use including spectrophotometer testing confirming a high UV absorption for the administered oestrogen compound^17,18^ and findings of oestrogen receptors in melanoma lesions^19,20^ have motivated a number of epidemiological studies examining both endogenous oestrogen exposure (e.g. age at menarche, parity) and exogenous oestrogen use (e.g., oral contraceptives [OCs], menopausal hormone therapy [MHT]).

Epidemiological studies evaluating the associations between oestrogen-related factors and melanoma risk have been inconsistent. A 2011 meta-analysis examining a variety of reproductive factors and exogenous hormone use found few pooled associations between melanoma and most oestrogen-related factors.^21^ However, increased melanoma risk was reported for older age at first birth, while decreased risk was found for parity, findings which could potentially be explained by reduced sun exposure among women with children.^21^ Most of the epidemiological studies to date have been either case–control studies^21,22^ or cohort studies in countries with relatively low levels of ambient UVR.^21,23,24^ In addition, few studies have examined whether oestrogen-related factors modify the relationship between UVR exposure and melanoma.^23,25^

Here we assess the associations between reproductive factors, exogenous oestrogen use and subsequent risk of melanoma among female participants of the National Institutes of Health (NIH)-AARP Diet and Health Study. To our knowledge, this is the largest prospective study to evaluate a broad range of reproductive factors and exogenous oestrogen use in relation to melanoma among US women exposed to substantial variation in ambient UVR.

## Materials and methods

### Overview

Detailed descriptions of the NIH-AARP Diet and Healthy Study cohort and methods have been previously published.^26^ Briefly, a self-administered questionnaire was sent to 3.5 million AARP members aged 50–71 years in six states (California, Florida, Pennsylvania, New Jersey, North Carolina, and Louisiana) and two metropolitan areas (Atlanta, Georgia and Detroit, Michigan) between 1995 and 1996. The baseline questionnaire gathered information on demographic characteristics, health-related behaviours and dietary intake. A second, risk factor questionnaire was administered between 1996 and 1997, collecting more detailed information on MHT use. The NIH-AARP study was approved by the Special Studies Institutional Review Board of the National Cancer Institute.

### Study population

Our study population included female, non-Hispanic white, postmenopausal participants of the NIH-AARP Diet and Healthy Study. Of the 566,398 participants who completed the baseline questionnaire, we serially excluded those with questionnaires filled out by proxies (*n* = 15,760), male respondents (*n* = 325,171), participants who died or were diagnosed with any cancer (excluding nonmelanoma skin cancer) before entry (*n* = 25,428), non-white participants (*n* = 21,867), women who were premenopausal at baseline (*n* = 9569), participants whose periods stopped due to radiation or were aged <60 years with no listed reason for menopause (*n* = 867), individuals without person-time at risk (*n* = 11) and individuals with missing information on UVR (*n* = 222). The resulting analytic cohort consisted of 167,503 participants. We also examined the associations between melanoma risk and detailed MHT use among a subset of women who also responded to the second questionnaire (*n* = 108,295).

### Cohort follow-up and case ascertainment

Participants were followed from their age at the completion of the baseline questionnaire (or the second risk factor questionnaire for analyses of detailed MHT use) until age of the earliest of cancer diagnosis, death, most recent linkage with cancer registries (December 31, 2011) or age when the participant moved out of the registry area. Incident melanoma cases were identified via probabilistic record linkage with state cancer registries and defined using the International Classification of Disease for Oncology, Third edition by anatomic site (C44.0-C44.9) and histology (8720-8780). This classification excludes melanomas described as metastases. Melanoma in situ and invasive melanoma were treated as separate outcomes and women with an incident melanoma in situ were censored in analyses for invasive melanoma. Invasive melanoma will henceforth be referred to simply as melanoma.

### Exposure and covariate assessment

Reproductive factors and exogenous oestrogen use were ascertained from the baseline questionnaire, which additionally collected information on education, marital status, body mass index (BMI), physical activity in the last 12 months, physical activity during adolescence (ages 15–18 years), smoking status, family history of cancer, age at menarche, parity, age at first live birth, menopausal reason, age at menopause, OC use and duration, MHT use and duration and status of surgical ovary removal. The follow-up risk factor questionnaire ascertained information on receiving a colonoscopy or sigmoidoscopy in the previous 3 years and detailed information on MHT use including oestrogen/progestin vs oestrogen only, dose, and duration.

Ground-level ambient UVR exposure for participants was assigned via linkage of geocoded residential location at study baseline to the Total Ozone Mapping Spectrometer database maintained by the National Aeronautics and Space Administration.^27^^,^^28^ This database provides daily estimates of noon-time erythemal UVR exposure on a 1° latitude by 1.25° longitude grid. Ground-level erythemal exposure was averaged across all available measured days in the month of July between 1978–1993 and 1996–2005, as levels of ambient UVR have remained relatively stable over time^29^ and surface UVR is strongest during the summer.^30^

### Statistical analysis

To assess the relationship between reproductive factors, exogenous oestrogen use and first primary melanoma, hazard ratios (HRs) and 95% confidence intervals (CIs) were computed using Cox proportional hazard regression analyses with age as the timescale. Follow-up started at age of entry into the cohort. The following variables were considered potential confounders because they could be significantly associated with both reproductive factors/exogenous oestrogen use and melanoma incidence but not believed to be on the causal pathway: birth cohort, education, marital status, BMI, smoking history, alcohol consumption, physical activity, coffee consumption, family history of cancer, colonoscopy/sigmoidoscopy in past 3 years, OC/MHT use (for reproductive factors), and ambient UVR. Although no direct measure of skin cancer screening was available in our data, age, education, marital status and colonoscopy/sigmoidoscopy in the past 3 years were examined as correlate of health-care utilisation to account for possible differences in medical surveillance. We also examined whether mutual adjustment for reproductive factors and exogenous hormones changed coefficients. Adjusted models included potential confounders that changed the risk coefficients by at least 15%.

Final models for reproductive factors were adjusted for age, education (less than high school, high school, some college, college, or graduate school), BMI (>18.5–<25, 25–<30, 30–<60 kg/m^2^), smoking status (never, former, current), marital status (married, widowed, divorced/separated, never married), family history of cancer (no, yes), colonoscopy/sigmoidoscopy (no, yes), MHT use (no, yes) and ambient UVR quartile (coded continuously 1–4). Final models for exogenous hormones were adjusted for age, education, BMI, smoking status (never, former, current), marriage (married, widowed, divorced/separated, never married), family history of cancer, colonoscopy/sigmoidoscopy, ever use of MHT (for OC models) and ambient UVR quartile. Missing or extreme values for covariates were coded as separate categories and included as indicator variables in the models. To address the hypothesis that oestrogen-related factors are photosensitising, ambient UVR was tested for multiplicative interaction with reproductive factors and exogenous hormone use.

We conducted several additional analyses. We investigated the association between melanoma risk and detailed MHT use among a subset of women who also responded to the second survey. We also examined whether updating information on MHT use to women who responded to the second survey using a time-dependent variable for MHT (never, past, current) impacted our findings. To assess the potential impact of medical surveillance/skin cancer screening, we explored the relationship between reproductive factors, exogenous oestrogen use and risk of melanoma in situ. The proportional hazards assumption was satisfied for all hazard models. Statistical tests were two-sided with a significance level of *α* = 0.05. Analyses were conducted using the SAS 9.4 software (SAS Institute, Cary, NC).

## Results

The study population included 167,503 non-Hispanic white, postmenopausal women who were cancer free at baseline. Over a median follow-up time of 15.5 years, 0.6% of eligible participants had an incident melanoma (*n* = 1061). Women with melanoma were of a similar age at study entry as women without melanoma (Table 1). A greater proportion of women with melanoma reported engaging in physical activity three or more times per week than women without melanoma, both in the past 12 months or during adolescence.

**Table 1:** Distribution of selected characteristics of women with and without melanoma in the NIH-AARP Diet and Health Study

Characteristic	% of women	
	No melanoma (*n* = 165,651)^a^	Melanoma (*n* = 1061)
Age at entry, years		
Mean	62.2	62.6
SD	5.3	5
Person-years at risk		
Mean	13.8	7.8
SD	3.9	4.4
Education^b^		
Less than high school	6	3
High school graduate	26.6	24.1
Some college	35.7	36.1
College or graduate school	29	33.7
Missing	2.8	3
Marital status		
Married	44.9	46
Widowed	23.3	22.7
Divorced/separated	24.8	24.9
Never married	6.5	5.8
Missing	0.7	0.7
Body mass index, kg/m^2 b^		
Normal, >18.5–<25	42.3	46.3
Overweight, 25–<30	31.5	33.6
Obese, 30–<60	21.8	18.2
Missing or extreme	4.5	2
Physical activity in the past 12 months^b^		
Never/rarely	22.4	17.3
1–3 times/month	14.3	14.7
1–2 times/week	21.1	20.8
3–4 times/week	24.9	27.8
≥5 times/week	16.4	18.4
Missing	0.9	0.9
Physical activity ages 15–18 years		
Never/rarely	16.5	13.9
1–3 times/month	8.9	8
1–2 times/week	17.6	18.9
3–4 times/week	24.1	25.5
≥5 times/week	32.1	32.9
Missing	0.6	0.8
Smoking status^b^		
Nonsmokers	43.4	44.8
Former	38.9	41.5
Current	14.6	10.2
Missing	3	3.6
Family history of cancer		
No	43.3	41.9
Yes	52.2	54
Missing	4.6	4.1
Colonoscopy/sigmoidoscopy in the past 3 years^c^		
No	42.9	44.3
Yes	20.9	21.5
Missing	36.2	34.2
Ambient UVR quartile, J/m^2^		
Q1 (176–186)	25.1	22.9
Q2 (187–239)	24.5	22.7
Q3 (240–253)	22.7	23.7
Q4 (254–290)	27.7	30.7

*UVR* ultraviolet radiation, *NIH* National Institutes of Health

^a^Excludes 791 cases of melanoma in situ

^b^Denotes *P* < 0.05 according to Chi-square

^c^Screening history was ascertained from second survey with <2% missing

We found an increased risk of melanoma among women who experienced menarche at an early age (age ≤10 vs ≥15 years: HR = 1.25, 95% CI: 0.92, 1.71; *P* for trend = 0.04), but no associations between age at first live birth or parity (Table 2). Late age at menopause increased melanoma risk (age ≥50 vs <45: HR = 1.34, 95% CI: 1.13, 1.59; *P* for trend = 0.001). When we stratify women by menopausal reason, we found a positive relationship between both age at natural menopause and melanoma risk (age ≥50 vs 40–44 years: HR = 1.30, 95% CI: 0.94, 1.79; *P* for trend = 0.01) and age at surgical menopause and melanoma risk (age ≥50 vs 40–44 years: HR = 1.11, 95% CI: 0.85, 1.47; *P* for trend = 0.005). Use of exogenous oestrogen or duration of use (at baseline) was not associated with melanoma (Table 3). These findings did not change when updating MHT use among women who also responded to the second survey. Analyses using detailed information on formulation of MHT collected on the second survey (i.e. oestrogen/progestin) also did not reveal any associations (Supplementary Table 1).

**Table 2:** Reproductive factors and risk of melanoma among women in the NIH-AARP Diet and Health Study

Factor	No. of woman-years^a^	No. of cases^a^	HR^b^ (95% CI)	*P* for trend^c^
Age at menarche^d^				
≥15 years	206,674	87	Reference	
13–14 years	960,120	423	1.03 (0.81–1.29)	
11–12 years	965,658	461	1.14 (0.91–1.44)	
≤10 years	151,591	75	1.25 (0.92–1.71)	0.04
No. of live births				
Nulliparous	339,783	167	1.24 (1.00–1.53)	
1–2	826,809	357	Reference	
≥3	1,115,914	524	1.08 (0.95–1.24)	0.75
Age at first live birth among parous women				
<20 years	375,890	133	Reference	
20–29 years	1,431,289	689	1.22 (1.01–1.48)	
≥30 years	129,880	57	1.08 (0.78–1.48)	0.25
Menopausal reason				
Natural menopause	1,277,438	569	Reference	
Surgical menopause	1,005,338	473	1.07 (0.93–1.21)	
Age at menopause^e^				
<45 years	771,682	314	Reference	
45–49 years	577,132	263	1.19 (1.00–1.42)	
≥50 years	930,360	467	1.34 (1.13–1.59)	0.001
Age at natural menopause				
<40 years	26,596	6	0.66 (0.28–1.55)	
40–44 years	118,110	42	Reference	
45–49 years	354,837	142	1.11 (0.79–1.57)	
≥50 years	773,958	376	1.30 (0.94–1.79)	0.01
Age at surgical menopause				
<40 years	377,003	143	0.79 (0.62–1.01)	
40–44 years	248,521	122	Reference	
45–49 years	220,837	120	1.09 (0.85–1.40)	
≥50 years	153,451	87	1.11 (0.85–1.47)	0.005
Ovary status, among surgical menopause				
Both removed	530,124	262	Reference	
Both intact	382,470	174	0.92 (0.76–1.15)	

*CI* confidence interval, *HR* hazard ratio, *NIH* National Institutes of Health

^a^Includes 167,503 women and 2,299,578 total woman-years, but numbers may be inconsistent because of missing values

^b^Adjusted for age, ambient ultraviolet radiation quartile (coded continuously 1–4), education (less than high school, high school, some college, college or graduate school, body mass index (>18.5–<25, 25–<30, 30–<60 kg/m^2^), smoking status (never, former, current), marriage (married, widowed, divorced/separated, never married), family history of cancer (no, yes), colonoscopy or sigmoidoscopy (no, yes), menopausal hormone therapy (no, yes)

^c^Trend tests were conducted by modeling ordinal categories as continuous

^d^Adjusted for factors mentioned in Table footnote b and menopausal age (<45, 45–49, 50+ years)

^e^Adjusted for factors mentioned in Table footnote b and menopausal reason (natural menopause, surgical menopause)

**Table 3:** Exogenous oestrogen use and risk of melanoma among women in the NIH-AARP Diet and Health Study

Factor	No. of woman-years^a^	No. of cases^a^	HR^b^ (95% CI)	*P* for trend^c^
OC use				
Never or <1 year	1,386,881	635	Reference	
Ever-users	888,852	408	1.02 (0.89–1.16)	
Duration of OC use				
Never or <1 years	1,386,881	635	Reference	
1–4 years	394,284	166	0.95 (0.80–1.13)	
5–9 years	278,949	133	1.06 (0.87–1.28)	
≥10 years	215,619	109	1.09 (0.88–1.34)	0.41
MHT use^d^				
Never	1,034,410	458	Reference	
Ever	1,260,650	599	1.02 (0.89–1.15)	
Past	212,649	91	0.92 (0.74–1.16)	
Current	1,048,001	508	1.04 (0.91–1.18)	
MHT duration^d^				
Never	987,164	437	Reference	
<5 years	440,939	188	0.95 (0.80–1.13)	
5–9 years	317,187	163	1.10 (0.92–1.33)	
≥10 years	504,234	248	1.01 (0.86–1.18)	0.68
MHT in women with natural menopause^d^				
Never	767,318	334	Reference	
Ever	508,536	234	0.99 (0.83–1.18)	
Past	107,336	36	0.72 (0.51–1.02)	
Current	401,200	198	1.06 (0.88–1.28)	
MHT duration^d^				
Never	744,033	329	Reference	
<5 years	253,718	103	0.90 (0.72–1.13)	
5–9 years	150,995	76	1.06 (0.82–1.36)	
≥10 years	104,202	55	1.01 (0.76–1.35)	0.87
MHT in women with surgical menopause^d^				
Never	253,152	110	Reference	
Ever	749,457	361	1.04 (0.83–1.29)	
Past	104,671	54	1.14 (0.82–1.58)	
Current	644,786	307	1.02 (0.81–1.28)	
MHT duration^d^				
Never	239,830	106	Reference	
5 years	186,471	83	0.98 (0.73–1.31)	
5–9 years	165,346	85	1.09 (0.82–1.47)	
≥10 years	398,876	193	0.99 (0.77–1.26)	0.99
MHT and OC use^d^				
Neither or <1-year OC/MHT use	709,283	304	Reference	
MHT use only	675,185	329	1.07 (0.91–1.25)	
OC use only	309,199	137	1.07 (0.87–1.31)	
OC and MHT use	577,907	270	1.06 (0.89–1.26)	

*CI* confidence interval, *HR* hazard ratio, *OC* oral contraceptive, *MHT* menopausal hormone therapy, *NIH* National Institutes of Health

^a^Includes 167,503 women and 2,299,578 total woman-years, but numbers may be inconsistent because of missing values

^b^Adjusted for age, ambient ultraviolet radiation quartile (coded continuously 1–4), education (less than high school, high school, some college, college or graduate school, body mass index (>18.5–<25, 25–<30, 30–<60 kg/m^2^), smoking status (never, former, current), marriage (married, widowed, divorced/separated, never married), family history of cancer (no, yes), colonoscopy or sigmoidoscopy (no, yes), menopausal hormone therapy (no, yes)

^c^Trend tests were conducted by modeling ordinal categories as continuous

^d^Adjusted for factors mentioned in Table footnote b except for menopausal hormone therapy (no, yes)

**Fig. 1:**
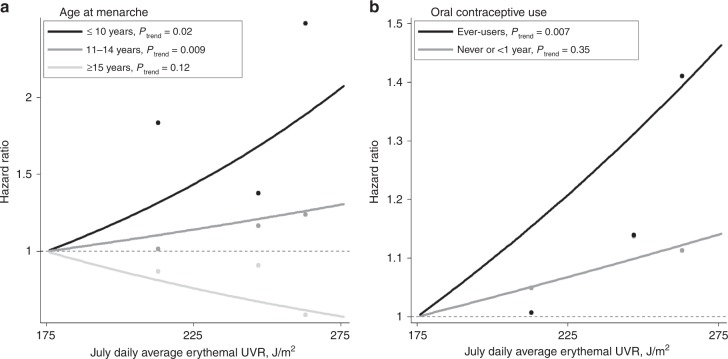
Risk of melanoma among non-Hispanic white, postmenopausal women according to ultraviolet radiation (UVR) exposure and **a** age at menarche and **b** oral contraceptive (OC) use. Solar ambient UVR points represent quartiles of exposure (J/m^2^). Trend and interaction *P* values are based on UVR quartile (coded continuously 1–4). *P* for interactions are 0.02 for age at menarche and 0.09 for OC use. Lines plotted using continuous UVR

The association between ambient UVR and melanoma risk was modified by age at menarche (*P* for interaction = 0.02), with the strongest association observed among women with early age at menarche (Fig. 1).

The relationship between UVR exposure and melanoma incidence was non-significantly higher among ever OC users (*P* for interaction = 0.09). The effect of UVR on melanoma incidence was not modified by age at menopause or MHT use (Supplementary Table 2).

To assess the sensitivity of our findings to the potential impact of skin cancer screening, we also examined the associations between oestrogen-related factors and risk of melanoma in situ. We found no associations between melanoma in situ and reproductive factors (Supplementary Table 3) or exogenous oestrogen use (Supplementary Table 4).

## Discussion

In this large cohort study, we prospectively evaluated the relationship between reproductive factors, exogenous oestrogen use and melanoma risk in US women residing in areas with substantial variation in ambient UVR. Early age at menarche and late age at menopause were associated with increased risk of melanoma. The association between ambient UVR and melanoma was strongest among women who experienced menarche at an early age. To our knowledge, this is the first study to demonstrate effect modification by age at menarche for the relationship between baseline ambient UVR and melanoma risk.

Melanoma risk was associated with early age at menarche and late age at menopause, supporting the hypothesis that oestrogen exposure is photocarcinogenic.^17,31^ Endogenous oestrogen levels are highest during the ovulation period, which begins at menarche and decreases sharply during the menopausal transition.^32^ Our results are consistent with another cohort study which found that shorter lifetime duration of ovulation was associated with decreased risk of melanoma among women living in France.^25^ However, a meta-analysis consisting primarily of case–control studies found no association between age at menarche, age at menopause and melanoma risk.^21^

We found no relationship between parity or age at first live birth and melanoma risk. These findings are in contrast to a 2011 meta-analysis that reported lower parity and higher age at first birth to be associated with risk of melanoma.^21^ The authors of the meta-analysis proposed that their findings could be due to residual confounding by socioeconomic or lifestyle factors related to individual sun exposure.^21^ A later prospective cohort study in France found a marginally decreased risk of melanoma among women with >3 births.^25^ The authors noted that the association between high parity and melanoma risk was somewhat stronger among premenopausal women, who were not included in our analysis.^25^ A large prospective cohort study in Denmark confirmed associations between parity, younger age at first birth and decreased melanoma risk for both women and men, lending support to the explanation that these associations might reflect confounding by lifestyle or socioeconomic factors.^24^ Our null findings are based on models adjusted for a number of lifestyle and socioeconomic factors (e.g. BMI, physical activity and education) but do not include individual measures of sun exposure or time outdoors. In addition, while pregnancy is associated with increased oestrogen exposure, endogenous oestrogen exposure decreases from breastfeeding. A lack of information on breastfeeding limits our ability to further interpret these null results.

Exogenous oestrogen use was not associated with risk of melanoma in our cohort. This finding agrees with previous research including a meta-analysis of seven case–control studies and three cohort studies that demonstrated no increased melanoma risk associated with OC use.^21^ However, a large case–control study in the Netherlands reported that melanoma was associated with both OC and MHT use.^22^ A recent large, prospective study of Norwegian women also reported a positive association between oestrogen-only MHT and melanoma risk but no association with oestrogen and progestin MHT and melanoma.^23^ Our findings are consistent with the US Women’s Health Initiative clinical trial, which found no association between MHT use and melanoma among white, postmenopausal women aged 50–79 years (though based on just 95 cases).^33^

We are the first to report effect estimates between current residential UVR and melanoma risk across strata of various hormone-related factors. Notably, melanoma risks associated with ambient UVR were highest in women with early age at menarche and among women who used OCs. Melanoma risk is believed to be most strongly associated with early-life sun exposure.^7,34–37^ The findings of effect modification by age at menarche and OC use, but not age at menopause or MHT use, underpin the importance of early-life oestrogen exposure for melanoma risk, especially among women living in locations with high levels of ambient UVR. Unfortunately, our study only had access to ambient UVR at baseline and did not have a UVR measure for childhood residence. To our knowledge, only two previous studies examined effect modification between UVR and factors related to endogenous oestrogen and hormone use for melanoma.^23,25^ Kvaskoff et al. found no evidence of interaction between endogenous oestrogens, ambient UVR and melanoma risk in a French cohort.^25^ Botteri et al. also reported that the association between MHT dose and melanoma risk did not vary across UVR levels in Norway.^23^ However, the US women in our large prospective study were exposed to a very broad range of ambient UVR.

This study has several limitations beyond those previously mentioned. We did not have access to a direct measure of skin cancer screening, which may induce a medical surveillance bias in analyses of prescribed hormone use and melanoma risk. Recently, increased screening (e.g. skin biopsy) has been linked to increases in diagnoses of melanoma in situ among the elderly US population.^38^ However, our findings of no association between exogenous hormone use or reproductive factors and melanoma in situ do not suggest a strong influence of medical surveillance/skin cancer screening bias in this cohort. Since the age of participants at the start of follow-up was relatively high (50–71 years), only late-onset melanoma could be examined. We also lacked information on pigmentation characteristics, including complexion, skin colour, eye colour, number of nevi and markers of genetic susceptibility. These risk factors were not likely strong confounders in the relationship between oestrogen-related factors and melanoma among non-Hispanic white women but cannot be ruled out as potentially important mediators of the association of photocarcinogenesis.

In summary, early age at menarche and late age at menopause were associated with increased risk of melanoma in this large, geographically dispersed cohort. The positive association between ambient UVR and melanoma risk was especially strong among women who experienced menarche at age ≤10 years and women who reported OC use, reinforcing the importance of early-life exposures for cutaneous melanoma. These finding suggest that women with longer cumulative exposure to endogenous oestrogens may benefit from more frequent skin cancer screening, especially those residing in areas with elevated levels of ambient UVR. Future studies should incorporate measures of personal lifetime UVR exposure, detailed sun-susceptibility information, information on breastfeeding and, ideally, longitudinal biological measurements of circulating oestrogens.

## Supplementary information

Supplementary Tables

## Data Availability

Materials and data are available from the corresponding author upon reasonable request.
