# Supplementary material for: Reproductive factors, exogenous hormone use and incidence of melanoma among women in the United States
Source: Br J Cancer. 2019 Feb 28;120(7):754–60. doi: 10.1038/s41416-019-0411-z (PMC6461881; doi:10.1038/s41416-019-0411-z)
Supplement: Supplementary file 1 — Supplementary Tables [file 41416_2019_411_MOESM1_ESM.docx]

### Supplementary Table 1. Detailed MHT Use and Risk of Melanoma Among Women in the NIH-AARP Diet and Health Study.

| **Factor** | **No. Woman-Years** | **No. cases^a^** | **HR^b^** | **95% CI** | ***P* for trend^c^** |
| --- | --- | --- | --- | --- | --- |
| Estrogen/progestin therapy^d^ |  |  |  |  |  |
| No HT | 569 247 | 271 | 1.00 | Referent |  |
| Ever | 427 797 | 209 | 0.99 | 0.89, 1.10 |  |
| Past | 166 941 | 80 | 0.94 | 0.73, 1.22 |  |
| Current | 252 960 | 123 | 0.95 | 0.76, 1.20 |  |
| Unknown | 7 896 | 6 | 1.49 | 0.66, 3.34 |  |
| Estrogen/progestin dose, among ever EPT^d^ |  |  |  |  |  |
| <1 mg | 16 990 | 11 | 1.28 | 0.68, 2.40 |  |
| 2.5 mg | 172 148 | 87 | 1.00 | Referent |  |
| 5 mg | 45 301 | 19 | 0.85 | 0.52, 1.40 |  |
| 10 mg | 35 796 | 16 | 0.89 | 0.52, 1.52 | 0.35 |
| Estrogen/progestin duration, among ever EPT^d^ |  |  |  |  |  |
| <5 years | 198 630 | 91 | 1.00 | Referent |  |
| 5-9 years | 114 643 | 57 | 1.04 | 0.75, 1.45 |  |
| ≥10 years | 98 444 | 53 | 1.03 | 0.73, 1.45 | 0.85 |
| Estrogen/progestin regimen, among ever EPT^e^ |  |  |  |  |  |
| No HT | 569 247 | 271 | 1.00 | Referent |  |
| ET | 437 636 | 189 | 0.88 | 0.72, 1.06 |  |
| Sequential | 138 020 | 57 | 0.82 | 0.62, 1.10 |  |
| 15-25 D/M | 66 854 | 28 | 0.81 | 0.55, 1.21 |  |
| Continuous | 173 214 | 101 | 1.18 | 0.93, 1.50 |  |
| Unknown regimen | 49 708 | 23 | 0.92 | 0.60, 1.42 |  |
| Estrogen only therapy^d^ |  |  |  |  |  |
| No HT | 569 247 | 271 | 1.00 | Referent |  |
| Ever | 437 636 | 189 | 0.88 | 0.73, 1.07 |  |
| Past | 116 664 | 50 | 0.87 | 0.64,1.17 |  |
| Current | 316 473 | 139 | 0.90 | 0.73, 1.11 |  |
| Unknown | 4 499 | 0 |  |  |  |
| Estrogen only dose^d^ |  |  |  |  |  |
| 0.3 mg | 29 096 | 12 | 0.90 | 0.49, 1.63 |  |
| 0.625 mg | 224 549 | 104 | 1.00 | Referent |  |
| 1.25 mg | 59 312 | 26 | 0.99 | 0.64, 1.52 |  |
| Other | 31 141 | 12 | 0.87 | 0.48, 1.59 | 0.86 |
| Estrogen only duration^d^ |  |  |  |  |  |
| <10 years | 210 560 | 78 | 1.00 | Referent |  |
| ≥10 years | 220 419 | 109 | 1.25 | 0.93, 1.68 |  |
| Estrogen only regimen^d^ |  |  |  |  |  |
| Daily | 265 685 | 115 | 1.00 | Referent |  |
| Other^f^ | 151 247 | 67 | 0.96 | 0.71, 1.29 |  |

Abbreviations: HR, hazard ratio; MHT, menopausal hormone therapy; EPT, estrogen/progestin therapy.

a Includes 108,295 women and 1 502 029 total woman-years, but numbers may be inconsistent because of missing values.

b Adjusted for age, ambient UV radiation quartile (coded continuously 1 through 4), education (less than high school, high school, some college, college or graduate school, body mass index (>18.5 to <25, 25 to <30, 30 to <60), smoking status (never, former, current), marriage (married, widowed, divorced/separated, never married), family history of cancer (no, yes), colonoscopy or sigmoidoscopy (no, yes), menopausal hormone therapy (no, yes)

c Trend tests were conducted by modeling ordinal categories as continuous.

d Adjusted for (b) except for menopausal hormone therapy (no, yes).

e Sequential defined as EPT use for fewer than 15 days per month, 15-25 D/M defined as 15-25 days per month

f Other defined as ET use less frequent than daily use

### Supplementary Table 2. Effect Modification of UVR and Estrogen-Related Factors and Risk of Melanoma Among Women in the NIH-AARP Diet and Health Study.

| **Factor** | **HR^a^** | **95% CI** | ***P* for UVR trend**^b^ | ***P* for interaction**^b^ |
| --- | --- | --- | --- | --- |
| No. of live births |  |  |  |  |
| Nulliparous | 1.11 | 0.97, 1.27 | 0.20 |  |
| 1-2 | 1.06 | 0.97, 1.16 | 0.35 |  |
| ≥3 | 1.07 | 0.99, 1.15 | 0.07 | 0.79 |
| Age at menarche |  |  |  |  |
| ≥15 years | 0.86 | 0.71, 1.04 | 0.12 |  |
| 11-14 years | 1.08 | 1.02, 1.15 | 0.009 |  |
| ≤10 years | 1.29 | 1.05, 1.58 | 0.02 | 0.02 |
| Age at menopause |  |  |  |  |
| <45 years | 1.07 | 0.96, 1.18 | 0.23 |  |
| 45-49 years | 1.03 | 0.93, 1.15 | 0.66 |  |
| ≥50 years | 1.10 | 1.01, 1.19 | 0.02 | 0.56 |
| OC use |  |  |  |  |
| Never or <1 year | 1.04 | 0.97, 1.11 | 0.35 |  |
| Ever | 1.13 | 1.04,1.24 | 0.007 | 0.09 |
| MHT use^c^ |  |  |  |  |
| Never | 1.07 | 0.99, 1.16 | 0.10 |  |
| Ever | 1.06 | 0.98, 1.14 | 0.15 | 0.67 |

Abbreviations: UVR, ultraviolet radiation; HR, hazard ratio; OC, oral contraceptives; MHT, menopausal hormone therapy.

a Hazard ratio comparing the hazard of melanoma per increase in UVR quartile after adjustment for age, education (less than high school, high school, some college, college or graduate school, body mass index (>18.5 to <25, 25 to <30, 30 to <60), smoking status (never, former, current), marriage (married, widowed, divorced/separated, never married), family history of cancer (no, yes), colonoscopy or sigmoidoscopy (no, yes), menopausal hormone therapy (no, yes)

b UVR quartiles treated as continuous (1 through 4).

c Adjusted for (a) and menopausal age (<45, 45-49, 50+).

### Supplementary Table 3: Reproductive Factors and Risk of Melanoma *in situ* Among Women in the NIH-AARP Diet and Health Study.

| **Factor** | **No. Woman-Years^a^** | **No. cases^a^** | **HR^b^** | **95% CI** | ***P* for trend^c^** |
| --- | --- | --- | --- | --- | --- |
| Age at menarche^d^ |  |  |  |  |  |
| ≥15 years | 206 674 | 62 | 1.00 | Referent |  |
| 13-14 years | 960 120 | 349 | 1.18 | 0.90, 1.55 |  |
| 11-12 years | 965 658 | 332 | 1.16 | 0.88, 1.52 |  |
| ≤10 years | 151 591 | 46 | 1.10 | 0.75, 1.62 | 0.71 |
| Parity |  |  |  |  |  |
| Nulliparous | 339 783 | 126 | 1.22 | 0.96, 1.57 |  |
| 1-2 children | 826 809 | 269 | 1.00 | Referent |  |
| ≥3 children | 1 115 914 | 390 | 1.06 | 0.90, 1.24 | 0.60 |
| Age at first live birth among parous women |  |  |  |  |  |
| <20 years | 375 890 | 95 | 1.00 | Referent |  |
| 20-29 years | 1 431 289 | 509 | 1.08 | 0.86,1.36 |  |
| ≥30 years | 129 880 | 53 | 1.15 | 0.81, 1.63 | 0.41 |
| Menopausal reason |  |  |  |  |  |
| Natural menopause | 1 277 438 | 458 | 1.00 | Referent |  |
| Surgical menopause | 1 005 338 | 327 | 0.89 | 0.77, 1.04 |  |
| Age at menopause^e^ |  |  |  |  |  |
| <45 years | 771 682 | 249 | 1.00 | Referent |  |
| 45-48 years | 577 132 | 188 | 0.93 | 0.76,1.13 |  |
| ≥49 years | 930 360 | 349 | 0.99 | 0.80, 1.19 | 0.91 |
| Age at natural menopause |  |  |  |  |  |
| <40 years | 26 596 | 6 | 0.51 | 0.22, 1.18 |  |
| 40-44 years | 118 110 | 54 | 1.00 | Referent |  |
| 45-49 years | 354 837 | 112 | 0.68 | 0.49, 0.93 |  |
| ≥50 years | 773 958 | 286 | 0.74 | 0.55, 0.99 | 0.54 |
| Age at surgical menopause |  |  |  |  |  |
| <40 years | 377 003 | 118 | 1.24 | 0.92,1.66 |  |
| 40-44 years | 248 521 | 70 | 1.00 | Referent |  |
| 45-49 years | 220 837 | 75 | 1.17 | 0.84, 1.61 |  |
| ≥50 years | 153 451 | 62 | 1.27 | 0.90,1.79 | 0.86 |
| Ovary status, among surgical menopause |  |  |  |  |  |
| Both removed | 530 124 | 178 | 1.00 | Referent |  |
| Both intact | 382 470 | 130 | 1.03 | 0.82, 1.30 |  |

Abbreviations: HR, hazard ratio.

a Includes 167 503 women and 2 299 578 total woman-years, but numbers may be inconsistent because of missing values.

b Adjusted for age, ambient UV radiation quartile (coded continuously 1 through 4), education (less than high school, high school, some college, college or graduate school, body mass index (>18.5 to <25, 25 to <30, 30 to <60), smoking status (never, former, current), marriage (married, widowed, divorced/separated, never married), family history of cancer (no, yes), colonoscopy or sigmoidoscopy (no, yes), menopausal hormone therapy (no, yes)

c Trend tests were conducted by modeling ordinal categories as continuous.

d Adjusted for (b) and menopausal age (<45, 45-49, 50+).

e Adjusted for (b) and menopausal reason (natural menopause, surgical menopause).

### Supplementary Table 4: Exogenous Estrogen Use and Risk of Melanoma *in situ* Among Women in the NIH-AARP Diet and Health Study.

| **Factor** | **No. Woman-Years^a^** | **No. cases^a^** | **95% CI** | ***P* for trend^c^** |
| --- | --- | --- | --- | --- |
| OC use |  |  |  |  |
| Never or <1 year | 910 211 | 471 | Reference |  |
| Ever | 578 026 | 313 | 1.11 (0.95 to 1.29) |  |
| Duration of OC use |  |  |  |  |
| Never or < 1 year | 910 211 | 471 | Reference |  |
| 1-4 years | 252 456 | 139 | 1.13 (0.93 to 1.37) |  |
| 5-9 years | 181 331 | 100 | 1.13 (0.91 to 1.41) |  |
| ≥10 years | 144 239 | 74 | 1.03 (0.81 to 1.33) | 0.40 |
| MHT use^d^ |  |  |  |  |
| Never | 663 753 | 320 | Reference |  |
| Ever | 835 560 | 468 | 1.11 (0.96 to 1.29) |  |
| Past | 137 864 | 73 | 1.06 (0.82 to 1.36) |  |
| Current | 697 696 | 395 | 1.13 (0.97 to 1.32) |  |
| MHT duration^d^ |  |  |  |  |
| Never | 635 691 | 306 | Reference |  |
| <5 years | 287 349 | 160 | 1.17 (0.97 to 1.43) |  |
| 5-9 years | 211 147 | 133 | 1.27 (1.03 to 1.56) |  |
| ≥10 years | 338 080 | 176 | 0.98 (0.81 to 1.18) | 0.85 |
| MHT in women with natural menopause^d^ |  |  |  |  |
| Never | 499 139 | 247 | Reference |  |
| Ever | 343 495 | 210 | 1.16 (0.96 to 1.41) |  |
| Past | 72 108 | 44 | 1.16 (0.84 to 1.61) |  |
| Current | 271 388 | 166 | 1.16 (0.94 to 1.43) |  |
| MHT duration^d^ |  |  |  |  |
| Never | 484 475 | 236 | Reference |  |
| <5 years | 168 503 | 97 | 1.17 (0.92 to 1.50) |  |
| 5- 9 years | 102 988 | 73 | 1.36 (1.03 to 1.78) |  |
| ≥10 years | 72 281 | 41 | 1.00 (0.71 to 1.40) | 0.25 |
| MHT in women with surgical menopause^d^ |  |  |  |  |
| Never | 156 790 | 70 | Reference |  |
| Ever | 490 409 | 255 | 1.11 (0.84 to 1.46) |  |
| Past | 65 450 | 28 | 0.92 (0.60 to 1.43) |  |
| Current | 424 959 | 227 | 1.15 (0.87 to 1.51) |  |
| MHT duration^d^ |  |  |  |  |
| Never | 149 349 | 70 | Reference |  |
| <5 years | 118 514 | 62 | 1.15 (0.82 to 1.63) |  |
| 5-9 years | 107 642 | 60 | 1.19 (0.83 to 1.69) |  |
| ≥10 years | 264 961 | 133 | 0.96 (0.71 to 1.29) | 0.58 |
| MHT and OC use^d^ |  |  |  |  |
| Neither or <1 year OC use | 342 461 | 149 | Reference |  |
| MHT use only | 170 424 | 175 | 1.13 (0.93 to 1.36) |  |
| OC use only | 154 133 | 66 | 1.17 (0.92 to 1.48) |  |
| OC and MHT use | 171 295 | 148 | 1.21 (0.99 to 1.48) |  |

Abbreviations: HR, hazard ratio; OC, oral contraceptive; MHT, menopausal hormone therapy.

a Includes 167,503 women, but numbers may be inconsistent because of missing values.

b Adjusted for age, UVR continuous quartiles (176-186, 187-239, 240-253, 254-289), education (less than high school, high school, some college, college or graduate school, body mass index (>18.5 to <25, 25 to <30, 30 to <60), smoking status (never, former, current), marriage (married, widowed, divorced/separated, never married), family history of cancer (no, yes), colonoscopy or sigmoidoscopy (no, yes), and menopausal hormone therapy (no, yes).

c Trend tests were conducted by modeling ordinal categories as continuous.

d Adjusted for (b) except for menopausal hormone therapy.
